# O2a O-antigen type and sepsis in invasive *Klebsiella pneumoniae*: evidence of association and clustering with an ST11-K64/CRKP background in a retrospective cohort

**DOI:** 10.3389/fcimb.2026.1746246

**Published:** 2026-03-13

**Authors:** Mengyan Li, Minghao Gu, Hong Wang, Yi Sun, Shan Jiang, Xiangke Wang, Shengyao Wang, Mingyu Wang, Sensen Lv, Xiudi Han, Xuedong Liu

**Affiliations:** 1 Department of Respiratory and Critical Care Medicine, Qingdao Municipal Hospital, Qingdao, China; 2 School of Clinical Medicine, Shandong Second Medical University, Weifang, China; 3 State Key Laboratory of Microbial Technology, Microbial Technology Institute, Shandong University, Qingdao, China

**Keywords:** invasive infection, *Klebsiella pneumoniae*, lipopolysaccharide, O2a, sepsis

## Abstract

**Background:**

Invasive *Klebsiella pneumoniae* (KP) infections complicated by sepsis are often associated with substantial mortality, which may partly result from dysregulated host immune–inflammatory responses to bacterial components, including lipopolysaccharide. KP O-antigen types have gained increasing attention in epidemiological surveillance and vaccine development, yet their relationship with sepsis risk in invasive KP infection remains insufficiently evaluated, particularly given potential confounding by clonal background and antimicrobial resistance. Here, we integrated clinical data with pathogen genomic and phenotypic characteristics to identify factors associated with sepsis in invasive KP infection and to characterize the underlying clonal and resistance context.

**Methods:**

This retrospective cohort study screened all consecutive patients with culture-confirmed KP infection at Qingdao Municipal Hospital (November 2016–October 2023) and identified invasive cases based on isolation from normally sterile sites; only the first invasive episode per patient was included. Sepsis was defined according to Sepsis-3 criteria. Antimicrobial susceptibility testing was performed routinely, and resistance phenotypes (CRKP/ESBL/MDR) were derived from susceptibility results. All isolates underwent whole-genome sequencing (paired-end 150 bp) on the MGI Tech MGISEQ-2000 platform; serotypes, sequence types, and virulence determinants were inferred using Kleborate. Core-genome SNP phylogenies were reconstructed using Snippy with recombination filtered by Gubbins and maximum-likelihood inference by IQ-TREE. Multivariable logistic regression was used to identify factors associated with sepsis.

**Results:**

Among 127 patients with invasive KP infection, the incidence of sepsis was 55.1% (70/127). In prespecified multivariable logistic regression models, bloodstream infection, higher PCT levels, and lymphocyte counts <1×10^9/L were associated with higher odds of sepsis after multivariable adjustment. Regarding pathogen-related factors, a “replacement exposure” strategy was applied by entering O2a, K64, and the ST11-K64 clonal background separately; O2a (OR 6.777, 95% CI 1.118–41.070), K64 (OR 16.674, 95% CI 1.588–175.022), and ST11-K64 (OR 19.525, 95% CI 1.991–191.461) were each significantly associated with sepsis, and Firth’s penalized regression yielded directionally consistent results, supporting robustness under sparse-data/separation concerns. Cross-tabulation analyses further demonstrated strong aggregation between O2a and K64 as well as the ST11-K64 background (K64 present in 83.3% and ST11-K64 in 77.8% of O2a-positive isolates, versus 0.9% and 0% among O2a-negative isolates, respectively), indicating that O2a and K64 predominantly arose from a high-risk ST11-K64 clonal subgroup. Phylogenetic analysis showed that this ST11-K64 lineage largely overlapped with carbapenem-resistant *Klebsiella pneumoniae* (CRKP) and harbored selected virulence determinants (e.g., aerobactin and rmpA2), supporting its designation as a high-risk lineage with combined resistance and virulence features.

**Conclusion:**

In this retrospective cohort of invasive KP infections, O2a was associated with a higher likelihood of sepsis. Given the strong clustering of O2a with the ST11–K64 clonal background and carbapenem-resistant phenotypes, O2a is more likely to serve as a clinically observable adjunct marker of a high-risk clonal/resistance lineage. These findings suggest the potential incremental value of O2a for early risk stratification, which warrants further evaluation for informing sepsis surveillance and assessment of resistance risk.

## 1 Introduction

Sepsis is a life-threatening syndrome characterized by organ dysfunction stemming from a dysregulated host response to infection (Singer et al., 2016). Despite advances in medical care, the incidence and mortality associated with sepsis remain unacceptably high, representing a significant global health burden. In 2017, it was estimated that 48.9 million sepsis cases were reported worldwide, with a mortality rate of 22.5%, accounting for nearly one-fifth of all deaths (Rudd et al., 2020). The burden of sepsis varies by the site of infection and the causative pathogenic agents. Among these, Gram-negative bacteria are the primary pathogens, with *Klebsiella pneumoniae* (KP) ranked second (Zhang et al., 2024).

In recent years, the increasing prevalence of hypervirulent *Klebsiella pneumoniae* (HvKp) has attracted widespread attention. HvKp infections often present as invasive disease and may progress to septic shock, resulting in substantial mortality. Systematic reviews and meta-analyses have reported that septic shock occurs in approximately 26% of HvKp infections, with an overall mortality of 21%; notably, when hypervirulence converges with carbapenem resistance (carbapenem-resistant HvKp, CR-HvKp), the pooled mortality can exceed 50%, and the risk of death is more than 12-fold higher than that of carbapenem-susceptible HvKp (Nagendra et al., 2025b). Additional meta-analyses suggest an increasing trend of hospital-acquired HvKp and CR-HvKp, underscoring the need for earlier identification and risk stratification of high-risk patients (Nagendra et al., 2025a). Collectively, these trends emphasize the urgent need for clinically actionable strategies to enable early recognition and effective risk stratification of high-risk KP infections.

The pathogenicity of KP is due to determinants of multiple virulence and host responses. Cell-wall lipopolysaccharide (LPS) functions as a key virulence factor, activating macrophages, dendritic cells, and neutrophils, subsequently triggering inflammatory cascades that facilitate the development of sepsis. The complete LPS molecule comprises three structural domains: lipid A (the endotoxic center initiating innate immune responses), a conserved core oligosaccharide, and the O-specific polysaccharide (O-antigen conferring immunogenicity) (Ramachandran, 2014; Gao and Guo, 2018; Qin et al., 2024). Among the nine identified O-antigen serogroups, four serogroups (O1, O2a, O3b, O5) account for over 70% of clinical isolates. Notably, O2a predominates among multidrug-resistant (MDR) KP and is closely associated with resistance to *β*-lactams and carbapenems (Zhang et al., 2020; Wang et al., 2023).

Existing clinical studies suggest that certain high-risk lineages within carbapenem-resistant *K. pneumoniae* (CRKP), such as ST11-K64, are more frequently encountered in severe invasive infections and are associated with sepsis occurrence and adverse outcomes (Wu et al., 2021; Zhou et al., 2021). In contrast, direct evidence remains limited and systematic clarification is lacking regarding whether O-antigen types (e.g., O2a) have an independent association with sepsis and whether they show stable clustering with high-risk lineages such as ST11-K64 or with antimicrobial resistance phenotypes. Therefore, we enrolled an invasive KP infection cohort and integrated whole-genome sequencing data to evaluate the association between O2a and sepsis, and to characterize its co-occurrence/clustering patterns with ST11-K64 and resistance phenotypes, thereby exploring the potential utility of O2a for identifying high-risk clonal backgrounds and for clinically actionable risk stratification.

## 2 Materials and methods

### 2.1 Study design

**Figure 1:**
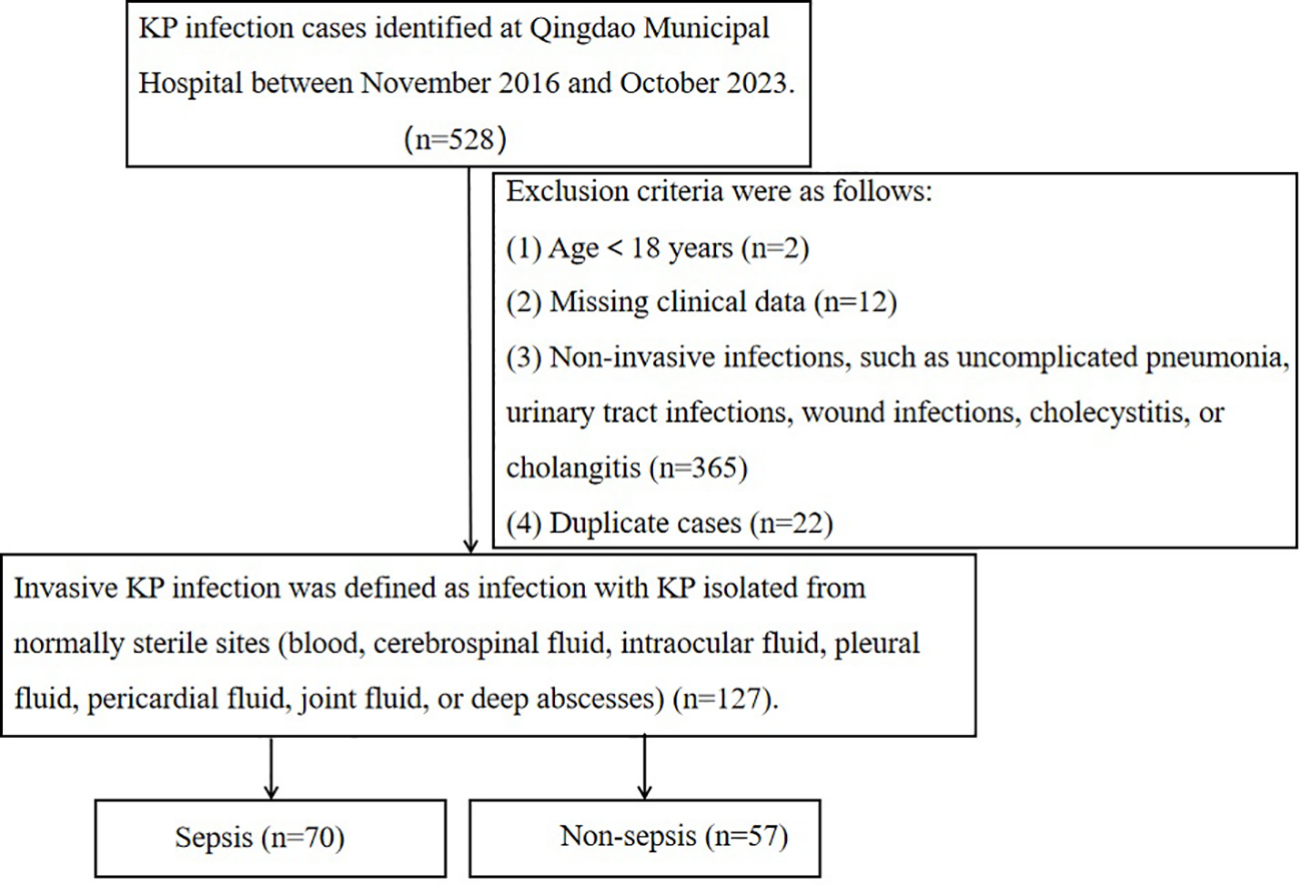
Flow chart for selection of study participants.

This retrospective cohort study was conducted at Qingdao Municipal Hospital, a tertiary facility located in Shandong Province, China. We captured all consecutive, culture-confirmed KP infections diagnosed between November 9, 2016, and October 15, 2023; all corresponding (non-duplicate) isolates underwent whole-genome sequencing (WGS), and patients with invasive KP infection were then identified from this cohort for subsequent analyses. Invasive infection was defined as KP isolated from normally sterile sites (e.g., blood, cerebrospinal fluid, intraocular fluid, pleural fluid, pericardial fluid, joint fluid, or deep abscesses). Non-invasive infections included pneumonia, urinary tract infections, wound infections, cholecystitis, cholangitis, and instances without documented bacteremia (Holt et al., 2015). Patients aged <18 years, those with missing key clinical data, or those with non-invasive infections were excluded. Only the first invasive episode per patient during the study period was included to ensure independence of observations. Sepsis was defined according to Sepsis-3 criteria as confirmed infection accompanied by an acute increase in SOFA score of ≥2 points from baseline. When pre-admission baseline SOFA was unavailable, baseline SOFA was assumed to be 0, and the SOFA score calculated within the first 24 hours of admission was used. In patients with documented chronic organ dysfunction, we estimated the baseline SOFA component(s) from the pre-infection clinical status and counted only the acute deterioration beyond baseline, while stable chronic impairments were not attributed to sepsis-related organ dysfunction (Singer et al., 2016). The study protocol was approved by the Ethics Committee of Qingdao Municipal Hospital (approval number: [XM:2023-030]). The research process is illustrated in **Figure 1**.

### 2.2 Data collection

Data were extracted from medical records using a pre-designed case report form. The following variables were recorded: demographic characteristics (age and sex); clinical features; intensive care unit (ICU) stay before infection onset; hospital-acquired infection (defined as infections occurring more than 48 hours post-hospital admission); comorbidities; invasive procedures prior to infection (arterial cannulation, central venous catheterization, invasive mechanical ventilation, urinary catheterization, gastric tube insertion, and surgical procedures within the preceding six months); treatment-related variables(Appropriate empirical antibiotic therapy; Time to appropriate antimicrobial therapy(hours); Source control); use of immunosuppressive agents (e.g., glucocorticoids, calcineurin inhibitors, antiproliferative agents, mechanistic target of rapamycin inhibitors, and biological agents) before infection; positive bacterial culture results; and concurrent laboratory parameters, including White blood cell count, Lymphocyte<1×10^9/L, Hemoglobin (g/L), hematocrit, procalcitonin (PCT), and other tests relevant to the diagnosis of sepsis; and 60-day all-cause mortality after infection.

### 2.3 Definitions

Appropriate empirical antibiotic therapy was defined as administration, within 24 hours after collection of the specimen yielding the index culture, of an antimicrobial regimen that included at least one agent with *in vitro* activity against the isolated KP. Time to appropriate antimicrobial therapy was defined as the interval (in hours) from collection of the specimen corresponding to the index culture to initiation of at least one antimicrobial agent with confirmed *in vitro* activity against the isolated strain, based on final susceptibility testing (Falcone et al., 2020). Source control was coded as a binary variable (yes/no) and defined as any documented procedural or surgical intervention aimed at eradication of the infectious focus, performed during the index episode (i.e., within 72 hours after index culture collection). Such interventions included abscess drainage, biliary decompression (e.g., ERCP or PTCD), surgical debridement, or removal of suspected infected intravascular devices (De Waele, 2024). CRKP was defined as non-susceptibility (I/R) to ≥1 carbapenem (e.g., imipenem and/or meropenem). MDR was defined as acquired non-susceptibility to at least one agent in three or more antimicrobial categories. ESBL-like phenotype was defined as extended-spectrum cephalosporin non-susceptibility (I/R) to ≥1 agent (e.g., ceftriaxone and/or cefotaxime and/or ceftazidime [± cefepime, depending on the panel]), without clavulanate-based confirmatory testing.

### 2.4 Antimicrobial susceptibility testing

Antimicrobial resistance phenotypes were established using the VITEK2 system (bioMérieux, Inc., Durham, NC, USA) and validated through broth microdilution and disk diffusion methods, with results interpreted according to Clinical and Laboratory Standards Institute (CLSI) guidelines. Exceptions were made for polymyxin and tigecycline: susceptibility to polymyxin was assessed in accordance with European Committee on Antimicrobial Susceptibility Testing (EUCAST) standards, while tigecycline susceptibility was interpreted according to FDA/NMPA guidelines. *Escherichia coli* ATCC 25922 was used as the quality control strain.

### 2.5 Whole-genome sequencing and bioinformatics

Genomic DNA was extracted from 1.2 mL of an overnight bacterial culture using the SPARKeasy Bacterial Genomic DNA Rapid Extraction Kit (Sikejie Biotech, Shandong, China) following the manufacturer’s protocol for Gram-negative bacteria and eluted in 66 μL deionized water. DNA concentration was measured using a micro-volume nucleic acid spectrophotometer (Nano-200) to ensure a total DNA amount ≥0.5 μg. DNA was stored at −20 °C (avoiding repeated freeze–thaw cycles) and transported on dry ice.

Library preparation was performed by using MGIEasy Universal DNA Library Prep Set (MGI-Shenzhen, China). Whole-genome sequencing was performed at BGI (Wuhan, China) using the MGISEQ-2000/DNBSEQ platform with paired-end 150-bp reads (PE150); clean FASTQ files were generated under the Phred+33 quality scoring system. Raw reads were processed using fastp (v0.23.4) for adapter trimming and quality control, with reads removed if they contained >1.0% ambiguous bases (N) or if ≥40.0% of bases had a Phred quality score <20. After filtering, per-isolate sequencing output typically yielded ~8.0 million clean read pairs and ~2.40–2.45 Gb clean bases, with high base quality (Q20 ~97.5–98.8% and Q30 ~92.5–96.2%). Coverage depth was estimated as clean bases divided by genome size; based on a typical K. pneumoniae genome size (~5–6 Mb), the theoretical mean sequencing depth was approximately >400× per isolate.

Clean reads were *de novo* assembled using SPAdes (v3.13.1). Assembly quality was summarized using QUAST (v5.0.2), including total length, GC content, number of contigs, and N50. Genome completeness and contamination were assessed using CheckM2 (v1.0.1), and completeness was further evaluated using BUSCO (v5.2.2). Species identity was verified using GTDB-Tk (v2.1.1), and only assemblies classified as Klebsiella pneumoniae were retained for downstream analyses.

Virulence determinants, MLST sequence types (STs), and capsule (K) and O-antigen (O) locus types (K-locus and O-locus) were inferred from genome assemblies using Kleborate (v2.3.2).

To support reproducibility, all software tools and versions are reported above, and the WGS data have been deposited in NCBI under BioProject accession PRJNA1205678.

### 2.6 Phylogenetic analysis

The KP genome with accession number SAMN02602959 was downloaded from NCBI as the reference genome. This reference genome was aligned with the assembled genomes from this study using Snippy v4.6.0 (https://github.com/tseemann/snippy.git) for single-nucleotide polymorphism (SNP) identification and assembly. Recombination was filtered using Gubbins v3.3.1. Maximum likelihood analysis was performed using IQ-Tree v2.3.6 software. All conserved polymorphic sites identified across isolates were concatenated for phylogenetic analysis. Finally, the phylogenetic tree was visualized using iTOL v7.0.

### 2.7 Statistical analysis

Continuous variables were summarized as mean ± standard deviation (SD) or median (interquartile range, IQR), as appropriate, and compared using Student’s t test or the Mann–Whitney U test. Categorical variables were presented as counts (percentages) and compared using the chi-square test or Fisher’s exact test. To characterize the aggregation among pathogen features (e.g., O-type, K-type, clonal background, and resistance phenotype), cross-tabulation analyses were performed and Cramer’s V was calculated to quantify the strength of association. Sepsis was used as the dependent variable in multivariable logistic regression. To evaluate model complexity and potential overfitting, events per variable (EPV) was conservatively calculated using the smaller outcome-group size divided by the number of model parameters included in each model. EPV was reported for the primary conventional models and for CRKP-adjusted sensitivity models. For binary predictors and continuous predictors, each variable contributes one parameter. Given the tight clustering among O2a, K64, ST11-K64, and resistance background, a prespecified “replacement exposure” strategy was applied: O2a, K64, and the ST11-K64 clonal background were entered separately under an identical covariate framework to mitigate severe collinearity and potential (quasi-) complete separation. The covariate framework included age, bloodstream infection (BSI), PCT, lymphocyte count < 1×10^9/L, and appropriate empirical antibiotic therapy, with additional adjustment for CRKP where applicable. Results were reported as odds ratios (ORs) with 95% confidence intervals (CIs). To assess robustness under sparse-data/separation concerns, Firth’s penalized likelihood logistic regression was further conducted as a sensitivity analysis; additional models incorporating both ST11-K64 and CRKP were also evaluated when appropriate. All tests were two-sided, and *P* < 0.05 was considered statistically significant.

## 3 Results

### 3.1 Clinical features of sepsis due to invasive KP infection

A total of 127 patients were enrolled in this study, with 55.12% (n = 70) in the sepsis group and 44.88% (n = 57) in the non-sepsis group. Compared with the non-sepsis group, patients in the sepsis group exhibited significantly higher PCT levels (*P* < 0.001), and lymphopenia (lymphocyte count < 1.0 × 10^9/L) was more frequent (*P* = 0.001). In addition, the sepsis group was more commonly associated with BSI and multiple infection sites (*P* < 0.05; **Supplementary Table 1**). The 60-day all-cause mortality following infection was also significantly higher in the sepsis group than in the non-sepsis group (*P* < 0.001; **Table 1**).

**Table 1:** Clinical characteristics of patients in the sepsis and non-sepsis groups.

Variables	Non-sepsis (n=57)	Sepsis (n=70)	P-value
Patients’ conditions			
Age, years	67 (56.00, 76.00)	71 (52.75, 81.00)	0.301
Male, n (%)	38 (66.67%)	50 (71.43%)	0.563
Underlying diseases, n (%)			
Diabetes mellitus	27 (47.37%)	29 (41.43%)	0.502
Chronic kidney diseases	7 (12.28%)	7 (10.00%)	0.683
Chronic lung diseases*	3 (5.26%)	2 (2.86%)	0.656
Cardiovascular diseases	31 (54.39%)	36 (51.43%)	0.740
Liver diseases	6 (10.53%)	7 (10.00%)	0.922
Biliary tract diseases	10 (17.54%)	14 (20.00%)	0.725
Cerebrovascular diseases	14 (24.56%)	19 (27.14%)	0.741
Malignant tumors and autoimmune diseases	8 (14.04%)	16 (22.86%)	0.207
Epidemiology, n(%)			
ICU	13 (22.81%)	25 (35.71%)	0.114
Hospital acquired	19 (33.33%)	31 (44.29%)	0.209
Laboratory results			
White blood cell (×10^9/L)	9.37 (6.70,12.06)	11.93 (6.53,18.57)	0.063
Lymphocyte <1×10^9/L	31(54.39%)	57(81.43%)	0.001
PCT (ng/mL)	2.76 (0.75,9.15)	12.60 (2.34,53.38)	<0.001
Hemoglobin (g/L)	105.00 (89.00,123.00)	106.00 (88.50,129.50)	0.769
Hematocrit (%)	32.10 (27.20,35.30)	32.20 (26.45,38.80)	0.479
Treatment measures, n (%)			
Arterial cannula/Central venous catheter	11 (19.30%)	23 (32.86%)	0.086
Invasive airway procedures	13 (22.81%)	15 (21.43%)	0.852
Gastric tube	16 (28.07%)	27 (38.57%)	0.214
Urinary catheter	16 (28.07%)	28 (40.00%)	0.160
Immunosuppressant therapy	5 (8.77%)	11 (15.71%)	0.241
Surgery	8 (14.04%)	10 (14.29%)	0.968
Treatment-related variables			
Appropriate empirical antibiotic therapy	51(89.47%)	57(81.43%)	0.206
Time to appropriate antimicrobial therapy from index culture collection, hours, median (IQR)	6.00(1.00,6.00)	6.00(1.50,7.75)	0.164
Source control	28(49.12%)	34(48.57%)	0.951
Infection type, n (%)			
BSI	34(59.65%)	64(91.43%)	<0.001
Liver abscess	25(43.86%)	20(28.57%)	0.073
Intra-abdominal infection*	1(1.75%)	7(10.00%)	0.073
Outcome, n (%)			
60-day mortality	1 (1.75%)	24 (34.29%)	<0.001

*Fisher’s exact test, ICU, intensive care unit; PCT, procalcitonin; BSI, Bloodstream infection.

### 3.2 Bacterial characteristics among patients in the sepsis and non-sepsis groups

In the microbiological comparison, isolates from the sepsis group showed a significantly higher prevalence of CRKP (*P* = 0.018), whereas the proportions of MDR and ESBL-like phenotypes did not differ significantly between groups (both *P* = 0.465). In terms of clonal distribution, ST11 was significantly enriched in the sepsis group (*P* = 0.003). Regarding O types, O1 was the most common type, followed by O2a, and the detection rate of O2a was significantly higher in patients with sepsis (*P* = 0.002). Among capsular (K) types, K1, K64, and K25 were relatively common, with K64 being markedly overrepresented in the sepsis group (*P* < 0.001). The distributions of virulence determinants were not statistically different between groups (**Table 2**).

**Table 2:** Distribution of antimicrobial resistance phenotypes, sequence types, K capsular types, O-antigen types, and virulence factors in invasive KP isolates by sepsis status.

Variables	Non-sepsis (n=57)	Sepsis (n=70)	P-value
CRKP, n (%)	9(15.79%)	24(34.29%)	0.018
MDR, n (%)	20(35.09%)	29(41.43%)	0.465
ESBL-like phenotype,n(%)	20(35.09%)	29(41.43%)	0.465
ST type, n (%)			
ST11	5(8.77%)	21(30.00%)	0.003
ST65*	2(3.51%)	4(5.71%)	0.690
ST23	18(31.58%)	15(21.43%)	0.195
O-antigen types, n (%)			
O1	33 (57.89%)	31 (44.29%)	0.127
O2a	2 (3.51%)	16 (22.86%)	0.002
O2afg*	6 (10.53%)	5 (7.14%)	0.540
O3b*	4 (7.02%)	5 (7.14%)	1.000
O5	4 (7.02%)	9 (12.86%)	0.280
Capsular types, n (%)			
K1	20 (35.09%)	18 (25.71%)	0.251
K2	7 (12.28%)	7 (10.00%)	0.683
K25	4 (7.02%)	8 (11.43%)	0.398
K64	1 (1.75%)	15 (21.43%)	<0.001
Virulence genes, n (%)			
Yersiniabactin*	41 (71.93%)	51 (72.86%)	0.907
Colibactin	19 (33.33%)	18 (25.71%)	0.347
Aerobactin	31 (54.39%)	45 (64.29%)	0.258
Salmochelin	32 (56.14%)	33 (47.14%)	0.313
RmpADC	32 (56.14%)	38 (54.29%)	0.834
rmpA2	29 (50.88%)	38 (54.29%)	0.702

*Fisher’s exact test, CRKP, carbapenem-resistant *Klebsiella pneumoniae*; MDR, multidrug-resistant; ESBL, extended-spectrum β-lactamase.

To contextualize the observed between-group differences, we further summarized antimicrobial resistance phenotypes across major O types, K types, and sequence types in the overall invasive cohort (**Supplementary Tables 2a–c**). Notably, O2a-positive isolates showed high rates of carbapenem resistance and multidrug resistance (CRKP, 15/18 [83.3%]; MDR, 16/18 [88.9%]), whereas O1 isolates exhibited substantially lower rates (CRKP, 2/64 [3.1%]; MDR, 9/64 [14.1%]). Consistent patterns were observed for K64 (CRKP, 14/16 [87.5%]; MDR, 15/16 [93.8%]) and ST11 (CRKP and MDR, 26/26 [100%]) (**Supplementary Tables 2b, c**), supporting a pronounced non-random co-occurrence between population structure and resistance profiles.

Cross-tabulation analyses further demonstrated strong aggregation between O2a and K64 as well as the ST11-K64 background: K64 was present in 83.3% of O2a-positive isolates (15/18) but in only 0.9% of O2a-negative isolates (1/109) (**Supplementary Table 3A**; Cramer’s V = 0.866), and the ST11-K64 combination occurred in 77.8% of O2a-positive isolates (14/18) while being absent among O2a-negative isolates (0/109) (**Supplementary Table 3D**; Cramer’s V = 0.866). In addition, carbapenem resistance was universal among ST11 isolates (CRKP in 100% of ST11-positive isolates, 26/26) (**Supplementary Table 3E**). Collectively, these findings indicate that O2a is not randomly distributed across the population structure but is tightly linked to a clonal background characterized by ST11-K64 with enrichment of carbapenem resistance; therefore, subsequent multivariable analyses employed prespecified “replacement exposure” models to evaluate O2a, K64, and ST11-K64 separately under the same covariate framework, thereby reducing the impact of severe collinearity and (quasi-)complete separation on estimate stability.

### 3.3 Risk factors for sepsis in invasive KP infection

In prespecified multivariable logistic regression analyses, we applied a “replacement exposure” strategy (O2a, K64, or ST11-K64 entered separately) under an identical covariate framework (age, BSI, PCT, lymphocyte count <1×10^9/L, and appropriate empirical antibiotic therapy; with additional adjustment for CRKP where applicable) to mitigate severe collinearity and (quasi-)separation arising from the tight aggregation among O-/K-type, clonal, and resistance backgrounds (**Supplementary Table 3**). In the primary conventional models, O2a was associated with increased odds of sepsis (OR 6.777, 95% CI, 1.118–41.070; *P* = 0.037), as were K64 (OR 16.674, 95% CI, 1.588–175.022; *P* = 0.019) and the ST11-K64 clonal background (OR 19.525, 95% CI, 1.991–191.461; *P* = 0.011), while BSI, PCT, and lymphocyte count <1×10^9/L also remained significant across models (all *P* < 0.05) (**Table 3**).

**Table 3:** Prespecified replacement multivariable logistic regression models for sepsis.

Variable	Model 1^a^		Model 2 ^b^		Model 3 ^c^	
	OR (95% CI)	P-value	OR (95% CI)	P-value	OR (95% CI)	P-value
age	1.018 (0.990–1.048)	0.214	1.020 (0.991–1.051)	0.180	1.021(0.992-1.051)	0.165
Appropriate empirical antibiotic therapy	3.454 (0.569–20.956)	0.178	3.055 (0.479–19.476)	0.237	2.121(0.532-8.451)	0.286
BSI	3.263 (1.019–10.449)	0.046	3.400 (1.057–10.932)	0.040	3.826(1.217-12.026)	0.022
Lymphocyte <1×10^9/L	4.714 (1.584–14.027)	0.005	4.070 (1.409–11.763)	0.010	4.049(1.431-11.454)	0.008
PCT	1.048 (1.019–1.079)	0.001	1.053 (1.023–1.085)	<0.001	1.053(1.022-1.085)	<0.001
CRKP	2.191 (0.467–10.269)	0.320	1.671 (0.350–7.974)	0.520	–	
O2a	6.777 (1.118–41.070)	0.037	–		–	
K64	–		16.674 (1.588–175.022)	0.019	–	
ST11-K64	–		–		19.525(1.991-191.461)	0.011

^a^Model 1: O2a + covariates (including CRKP).

^b^Model 2: K64 + covariates (including CRKP).

^c^Model 3: ST11-K64 clonal background + covariates (excluding CRKP).

Covariates included age, BSI, PCT, lymphocyte count <1×10^9/L, and appropriate empirical antibiotic therapy. O2a (Model 1), K64 (Model 2), and the ST11-K64 clonal background (Model 3) were entered separately (“replacement exposure” strategy). Models 1–2 were additionally adjusted for CRKP, whereas Model 3 excluded CRKP due to its near-complete overlap with ST11-K64. EPV (events per variable) was calculated as the number of non-sepsis events (n=57) divided by the number of predictors in each model: Accordingly, Model 3 included 6 predictors (EPV = 9.5, 57/6), whereas Models 1–2 included 7 predictors due to additional adjustment for CRKP (EPV = 8.14, 57/7).

BSI, Bloodstream infection; PCT, Procalcitonin; CRKP, Carbapenem-resistant *Klebsiella pneumoniae.*

To assess robustness under sparse-data conditions, Firth’s penalized logistic regression yielded directionally consistent and statistically significant associations for O2a (OR 5.121, 95% CI, 1.149–32.550; *P* = 0.031), K64 (OR 10.107, 95% CI, 1.751–113.780; *P* = 0.008), and ST11-K64 (OR 11.893, 95% CI, 2.207–128.193; *P* = 0.002) (**Supplementary Tables 4A–C**). Given the strong overlap between ST11 and carbapenem resistance, we further evaluated models incorporating CRKP: in a conventional model including both ST11-K64 and CRKP, ST11-K64 remained significant (OR 15.172, 95% CI, 1.368–168.262; *P* = 0.027) whereas CRKP was not (*P* = 0.539) (**Supplementary Table 4D**); similarly, in a Firth model with additional CRKP adjustment, ST11-K64 remained associated with sepsis (OR 9.315, 95% CI, 1.478–108.436; *P* = 0.016) while CRKP remained non-significant (*P* = 0.562) (**Supplementary Table 4E**).

### 3.4 Clinical features, virulence–resistance profiles, and phylogenomic clustering of O2a-positive KP

Among 127 patients with invasive KP infection, 109 had O2a-negative KP and 18 had O2a-positive KP (**Table 4**). Compared with infections caused by O2a-negative KP, O2a-positive infections were more likely to occur in patients already in the ICU before infection onset and were more frequently hospital-acquired (both *P* = 0.002). The distribution of infection type showed that almost all cases in the O2a-positive group presented with BSI (*P* = 0.012), with a higher proportion of intra-abdominal infection (*P* = 0.001), whereas liver abscess was not observed in the O2a-positive group (*P* < 0.001) (**Table 4**; **Supplementary Table 1**). In terms of clinical severity and outcomes, the O2a-positive group had higher SOFA scores (*P* < 0.001), a higher proportion with Sepsis (*P* = 0.002), a higher 60-day mortality after infection (*P* = 0.002), and a lower rate of appropriate empirical antimicrobial therapy (*P =* 0.001) (**Supplementary Tables 2**–**4**). In terms of microbiological characteristics, the O2a-positive group showed a marked enrichment of the ST11-K64 lineage (*P* < 0.001) and a substantially higher prevalence of resistance phenotypes, including CRKP (*P* < 0.001), MDR (*P* < 0.001), and an ESBL-like phenotype (*P* < 0.001).

**Table 4:** Clinical and Microbiological Characteristics of the O2a-positive vs O2a-negative infections.

Variables	O2a-positive (n=18)	O2a-negative (n=109)	*P* value
Patients’ conditions			
Age, years	67(44.25,75.75)	68(57.00,78.00)	0.230
Male, n (%)	12(66.67%)	76(69.72%)	0.794
Underlying diseases, n (%)			
Diabetes mellitus	8 (44.44%)	48 (44.04%)	0.974
Chronic kidney diseases*	4 (22.22%)	10 (9.17%)	0.113
Malignant tumors and autoimmune diseases*	4 (22.22%)	20 (18.35%)	0.746
Epidemiology, n (%)			
ICU	11 (61.11%)	27 (24.77%)	0.002
Hospital acquired	13 (72.22%)	37 (33.94%)	0.002
Infection type, n (%)			
BSI*	18(100%)	80(73.39%)	0.012
Liver abscess	0(0.00%)	45(41.25%)	<0.001
Intra-abdominal infection*	5(27.78%)	3(2.75%)	0.001
treatment-related variables			
Appropriate empirical antibiotic therapy*	10 (55.56%)	98 (89.91%)	0.001
Time to appropriate antimicrobial therapy from index culture collection, hours, median (IQR)	9.00(1.00,40.75)	6.00(1.00,6.00)	0.238
Source control	7(38.89%)	55(50.46%)	0.363
SOFA	4.5(3.00,7.00)	1.00(0.00,3.00)	<0.001
Sepsis	16(88.89%)	54(49.54%)	0.002
60-day mortality*	9(50.00%)	16(14.68%)	0.002
ST11-K64*	14(77.78%)	0(0.00%)	<0.001
CRKP*	15 (83.33%)	18 (16.51%)	<0.001
MDR	16(88.89%)	33(30.28%)	<0.001
ESBL-like phenotype	15 (83.33%)	34(31.19%)	<0.001

*Fisher’s exact test.

ICU, Intensive care unit; BSI, Bloodstream infection; SOFA, Sequential Organ Failure Assessment; CRKP, Carbapenem-resistant *Klebsiella pneumoniae*; MDR, Multidrug-resistant; ESBL, Extended-spectrum β-lactamase.

**Figure 2:**
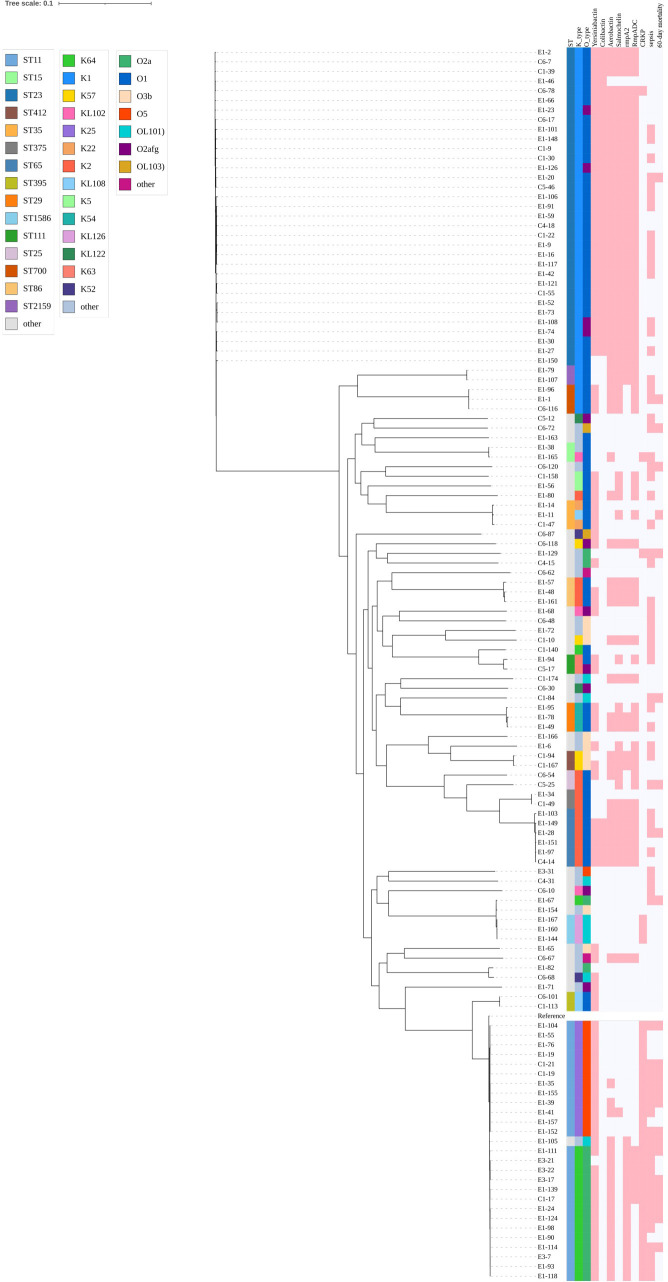
Phylogenetic clustering of O2a within the high-risk ST11-K64 clonal lineage. A whole-genome–based phylogenetic tree of *Klebsiella pneumoniae* isolates is shown, with branch lengths proportional to genetic distance (scale bar, 0.1). The colored annotation strips adjacent to the tree indicate in silico–predicted multilocus sequence type (ST), capsule locus (K type), and O-antigen type (O type), as defined in the legend. The right-hand binary presence/absence matrix summarizes the distribution of virulence loci/systems (yersiniabactin, colibactin, aerobactin, salmochelin, rmpADC and rmpA2), together with clinical/resistance phenotypes (CRKP, sepsis, and 60-day all-cause mortality). Pink denotes presence/”yes” and white denotes absence/”no”. Isolate identifiers are listed along the y-axis; a reference genome is included and labeled “Reference”. ST, sequence type; CRKP, carbapenem-resistant *K. pneumoniae*.

Furthermore, the core-genome phylogeny annotated with ST/K/O types and key resistance/virulence features showed that most O2a-positive isolates clustered within a closely related ST11-K64 lineage, with a concordant high frequency of carbapenem resistance and carriage of selected virulence determinants (e.g., rmpA2 and aerobactin). In this phylogeny, ST11-K64 isolates formed a tightly clustered clade with limited within-clade diversity, and this clade largely coincided with the distribution of O2a-positive isolates. Together with the cross-tabulation results (**Supplementary Table 3**), these findings support that O2a is not randomly distributed but primarily tracks a specific high-risk clonal background (**Figure 2**).

## 4 Discussion

This study analyzed a consecutive cohort of patients with invasive KP infection by integrating clinical characteristics, antimicrobial resistance phenotypes, and whole-genome sequencing data to explore factors associated with sepsis. Sepsis was defined according to Sepsis-3 criteria as documented infection accompanied by an acute increase in SOFA score of ≥2 points from baseline. We observed that O2a was significantly enriched in the sepsis group and showed a consistent risk direction across prespecified “replacement-exposure” multivariable models and Firth penalized logistic regression sensitivity analyses. Notably, O2a showed strong co-occurrence with K64, the ST11-K64 clonal background, and carbapenem resistance, indicating that this association is best interpreted within a clustered lineage/resistance context rather than as evidence for a standalone causal effect of O2a. Accordingly, O2a may serve as a pragmatic adjunct marker for surveillance and risk stratification when interpreted as part of a broader set of lineage and resistance markers (ST, K type, and CRKP phenotype) and should not be interpreted as a primary or exclusive indicator. Patients with O2a-positive infections also presented with greater illness severity and were more likely to receive discordant empirical therapy, which may partly reflect higher resistance burden and delayed effective coverage.

Consistent with previous reports, in our KP cohort, sepsis predominantly occurred in the setting of BSI and was characterized by higher PCT levels and more frequent lymphopenia. In the prespecified multivariable models, elevated PCT, reduced lymphocyte counts, and the presence of BSI remained independently associated with sepsis. Huang et al. similarly reported that increased PCT was independently related to sepsis among patients with HvKP infection (Huang et al., 2023). In addition, prior cohort studies have shown that persistent lymphopenia is closely linked to the greatest disease severity and the poorest clinical outcomes in sepsis, and is regarded as a key phenotypic hallmark of sepsis-associated immunosuppression; this aligns with the lymphopenia-related risk signal observed in our study (Huang et al., 2026). Notably, Chen et al. reported that secondary BSI following KP pneumonia was associated with an approximately threefold higher risk of developing sepsis (Chen et al., 2023). From a pathophysiological perspective, BSI is expected to increase systemic exposure to pathogens and pathogen-associated components, which may contribute to heightened innate immune activation and systemic inflammation; these processes are implicated in microcirculatory/endothelial dysfunction and organ injury in sepsis, although such mediators were not directly measured in our study (Szijártó et al., 2017; Wang et al., 2023).

Within the prespecified replacement-exposure framework, O2a, K64, and the ST11-K64 clonal background showed concordant directions of association with sepsis, consistent with their strong co-occurrence in our cohort. Prior reports from China have described ST11-K64 as being more common in ICU and hospital-acquired infections and associated with greater needs for invasive supportive care in CRKP populations (Wu et al., 2024; Li et al., 2025). Wu et al. further reported a higher short-term mortality risk among patients with ST11-K64 KP BSI (Wu et al., 2021), and Zhou et al. found that ST11-K64 was significantly associated with a higher incidence of sepsis in analyses of carbapenem-resistant Enterobacterales BSI (Zhou et al., 2021). Collectively, these data are consistent with our findings. In line with microbiological descriptions of ST11-K64 from multiple regions in China, we also observed frequent co-occurrence of ST11-K64 with carbapenem resistance and enrichment of virulence-associated loci (e.g., aerobactin and rmpA2) in our dataset (Zhang et al., 2022; Wang et al., 2024; Wu et al., 2024; Wang and Wang, 2025). Although functional hypervirulence was not directly assessed, this resistance–virulence locus co-occurrence is consistent with prior epidemiologic descriptions of ST11-K64 as a clinically high-risk lineage. In this context, the aggregation of O2a and K64 within ST11-K64 supports their use as adjunct antigenic tags for lineage tracking and surveillance when interpreted alongside lineage and resistance information.

Given the pronounced lineage–resistance clustering described above, O2a is best conceptualized as an auxiliary antigenic tag that annotates a high-risk clonal/resistance background rather than an independent pathogenic determinant. Because O-antigen diversity is generally lower than capsular (K) diversity and a limited number of O types account for most clinical isolates, O-type information may be particularly useful for region-specific stratified surveillance and early recognition in settings where high-risk lineages (e.g., ST11-K64/CRKP) are prevalent (Follador et al., 2016; Choi et al., 2020). Nevertheless, the incremental value of incorporating O2a into clinical or surveillance workflows should be evaluated in larger, multicenter cohorts with standardized phenotyping and external validation.

## 5 Limitations

This study has several limitations. First, the retrospective, single-center design may introduce selection bias and limits generalizability. Second, our statistical framework relied on prespecified modeling choices (including a replacement-exposure strategy and sensitivity analyses) that cannot fully eliminate residual confounding. Third, strong collinearity and co-occurrence among O2a, the ST11-K64 lineage, and carbapenem resistance constrained stable estimation of independent effects; therefore, our findings should be interpreted primarily as association and epidemiologic clustering rather than causality. Fourth, sepsis ascertainment followed Sepsis-3 (SOFA-based classification) using routinely recorded clinical and laboratory data, which may be affected by timing and documentation variability. Fifth, given the number of candidate predictors relative to events, model overfitting remains possible; although we monitored EPV (using the smaller outcome group divided by the number of model parameters) and performed sensitivity analyses, broader internal validation and external validation are needed. Sixth, although we captured several treatment-related variables (e.g., timeliness/appropriateness of antimicrobials and source control), the granularity and completeness of resuscitation and ICU-support data (e.g., fluid balance, vasopressor dose/duration, ventilation parameters) were limited. Overall, these constraints preclude causal inference, and prospective multicenter studies with standardized sepsis phenotyping and richer treatment data are warranted.

## 6 Conclusion

In invasive KP infections, O2a was associated with higher SOFA scores and a higher proportion of sepsis, and it showed tight clustering with the ST11–K64 lineage and carbapenem-resistant phenotypes. Collectively, these observations suggest that O2a may serve as a practical adjunct marker for risk stratification and lineage tracking, whose incremental value warrants further evaluation to support surveillance prioritization and assessment of resistance risk in settings where high-risk KP lineages are prevalent.

## Data Availability

The datasets presented in this study can be found in online repositories. The names of the repository/repositories and accession number(s) can be found in the article/**Supplementary Material**.

## Glossary

BSI: Bloodstream infection
CI: Confidence intervals
CLSI: Clinical and Laboratory Standards Institute
EUCAST: European Committee on Antimicrobial Susceptibility Testing
ICU: Intensive care unit
KP: *Klebsiella pneumoniae*
OR: Odds ratios
SOFA: Sequential Organ Failure Assessment
HvKp: Hypervirulent *Klebsiella pneumoniae*
CRKP: Carbapenem-resistant *Klebsiella pneumoniae*
MDR: Multidrug-resistant
ESBL: Extended-spectrum β-lactamase
CR-HvKp: Carbapenem-resistant Hypervirulent *Klebsiella pneumoniae*
